# Understanding the Tensions of “Good Motherhood” Through Women’s Digital Technology Use: Descriptive Qualitative Study

**DOI:** 10.2196/48934

**Published:** 2023-10-25

**Authors:** Danica Facca, Jodi Hall, Bradley Hiebert, Lorie Donelle

**Affiliations:** 1 Department of Health Information Science Faculty of Information and Media Studies Western University London, ON Canada; 2 School of Nursing Fanshawe College London, ON Canada; 3 Arthur Labatt Family School of Nursing Western University London, ON Canada; 4 College of Nursing University of South Carolina Colombia, SC United States

**Keywords:** motherhood, parenting, digital health, apps, social media, mother, parent, technology use, use, computer use, interview, interviews, perspective, perspectives, mothers, mobile phone

## Abstract

**Background:**

Research suggests that expectant and new mothers consult and value information gathered from digital technologies, such as pregnancy-specific mobile apps and social media platforms, to support their transition to parenting. Notably, this transitional context can be rich with profound physiological, psychological, and emotional fluctuation for women as they cope with the demands of new parenting and navigate the cultural expectations of “good motherhood.” Given the ways in which digital technologies can both support and hinder women’s perceptions of their parenting abilities, understanding expectant and new mothers’ experiences using digital technologies and the tensions that may arise from such use during the transition to parenting period warrants nuanced exploration.

**Objective:**

This study aims to understand mothers’ use of digital technologies during the transition to parenting period.

**Methods:**

A descriptive qualitative study was conducted in a predominantly urban region of Southwestern Ontario, Canada. Purposive and snowball sampling strategies were implemented to recruit participants who had become a parent within the previous 24 months. Researchers conducted focus groups using a semistructured interview guide with 26 women. The interviews were audio recorded, transcribed, and thematically analyzed.

**Results:**

Participants’ experiences of using digital technologies in the transition to parenting period were captured within the overarching theme “balancing the tensions of digital technology use in the transition to parenting” and 4 subthemes: self-comparison on social media, second-guessing parenting practices, communities of support, and trusting intuition over technology. Although digital technologies purportedly offered “in-the-moment” access to community support and health information, this came at a cost to mothers, as they described feelings of guilt, shame, and self-doubt that provoked them to question and hold in contention whether they were a good mother and using technology in a morally upright manner.

**Conclusions:**

These findings raise critical questions concerning the promotion and commercialization of digital technologies and the ways in which they can further push the boundaries of hegemonic parenting practices, provoke feelings of inadequacy, and compromise well-being among expectant and new mothers.

## Introduction

### Background

Expectant parents, predominantly mothers, regularly use internet-based resources such as websites, internet-based forums, and blogs for informational needs, access to services, and social support during the transition to parenting [1-4]. The transition to parenting period consists of 4 stages—pre-conception, pregnancy, labor, and postpartum—which uniquely mark the time in a person’s life when they become a parent for the first time or add another child to their family [1,4]. These stages will inevitably vary from person to person depending on their unique family dynamic and health circumstances [1,3,4]. Nonetheless, advances in digital technologies (eg, computers, web cameras, wearable technologies, smartphones, and internet-based applications), along with the introduction of social media in the mid-2000s, have expanded the technological landscape in which expectant and new parents gather information and connect with others to support their parenting practices throughout these nascent stages of parenthood [5]. During the transition to parenting, recent studies suggest that new and expectant mothers consult and value information gathered from digital technologies such as pregnancy-specific mobile apps and social media platforms such as YouTube to search for signs of normality and risks of illness and to find a maternal community [1,5]. Social media and pregnancy apps have been found to promote women’s well-being by reducing feelings of isolation and improving their own and their new or developing infants’ health outcomes by providing immediate access to medical information and how-to videos on infant care [1,6,7].

The proliferation of digital technologies, particularly within the health and medical sector, offers expecting parents endless opportunities and novel ways to use their personal digital devices to monitor, photograph, index, catalog, video record, and compare their maternal bodies to others in real time [8]. Within the transition to parenting period, comparing and evaluating one’s pregnant body and the developing fetus or newborn against those of other pregnant and fetal bodies has become a normalized parenting practice and way to “do pregnancy” [9]. In fact, pregnancy-related apps are the most-used health apps [10] and the number of pregnancy app downloads that offer expectant parents an avenue to self-monitor their bodies and that of their growing fetus continues to increase yearly across major app platforms [9]. For example, as of May 2023, Pregnancy +, the most popular pregnancy tracking app available on the Apple App Store and Google Play, has an estimated 50 million users worldwide [11]. Within the highly competitive commercialized app world, pregnancy apps are marketed specifically to cisgender women to document and benchmark their prenatal and early parenting practices as a taken-for-granted aspect of parental care and marker of being a “good mother” [9].

The widespread datafication and dataveillance [12] aligned with the ideals of good motherhood within North American culture encourages mothers’ development of self-knowledge and peace of mind through daily digital technology use; these practices simultaneously reinforce sociotechnical structures and systems that allow corporate entities such as app developers and technology conglomerates to track and mine data for enhanced business intelligence and performance [9]. Within this context, *datafication* refers to personal behaviors such as bodily movements, thoughts, and emotions that are monitored and quantified through digital interfaces to produce data that can be analyzed and explored to deepen our understanding of human behavior [12]. *Dataveillance*, on the other hand, is a concept that refers to the broader internet-based environment wherein datafication occurs and the ways in which users’ personal behaviors are constantly being watched and guided by the technical infrastructure they are interacting with [12].

### On Guilt in the Transition to Parenting

The transition to parenting is a period rich with profound physiological, psychological, and emotional fluctuations for individuals as they cope with the demands of new parenthood and navigate cultural expectations of successful parenting. Specifically, women are charged with the responsibility to take it upon themselves to enter the ranks of performing good motherhood [3,13]. As some research notes, North America’s cultural ideology of good motherhood asks women to give their all—physically, emotionally, psychologically, and intellectually—at all times, which consequently presents women with a model of nearly unachievable expectations [14]. Within the transition to parenting period, good motherhood in North America is associated with women showing unrelenting consideration, care, and love for their expectant or new infants by willingly engaging in vigilant self-care and information-seeking practices to ensure a healthy pregnancy, delivery, and optimal infant development during the early postpartum years [15].

With such demanding sociocultural expectations placed on new mothers, it is foreseeable that mothers regularly report feeling guilty when they do not or cannot exude the narrowly defined social standards of good motherhood, which positions White, heterosexual, cisgender, and middle-class mothers as normative [14,16,17]. In fact, maternal guilt is so pervasive in North American culture that it is considered an expected, almost inherent aspect of mothering norms by some scholars [14,18]. In psychological terms, a person experiences guilt—a negative evaluation of their own behavior or attitude—when they become conscious that they have wronged someone else; guilt involves criticizing one’s *actions specifically* [19]*.*

In this way, North America’s cultural expectations of good motherhood are inextricably linked to women’s “moral selves,” as they are expected to navigate a social system with purported “right” and “wrong” ways to mother [14,18,19]. For example, the “right” way to mother may involve having an unmedicated birth; breastfeeding; and feeling *only* joy, happiness, and gratitude for the privilege of becoming a mother during the postpartum period. Therefore, the “wrong” way to mother might consist of drug-involved labor (eg, the use of a narcotic for pain relief); formula feeding; and expressing feeling sad, angry, or otherwise disappointed following a new baby’s arrival [13]. The idealized good mother is thereby “involved,” always present and attentive to the needs of their infants or children, a constant model, guide, and teacher [20]. Through this lens, North American mothers are set up to experience constant maternal guilt in a cultural landscape that positions any shortcoming of meeting “right ways” to mother as a personal, moral failing.

In relation to digital technologies, experiences of guilt are amplified through the perpetual supply of curated content by other mothers [17]. Despite the potential benefits that pregnancy-specific apps and social media platforms offer mothers as tools for health information seeking and finding social support, the use of these technologies has been found to perpetuate feelings of guilt, shame, inadequacy, and self-doubt that are bound up within cultural expectations of good motherhood [17-19]. For example, research that focused on mothers’ use of digital technologies during the perinatal period found that they reported feelings of anxiety in relation to the developmental milestones of infants described in the apps [13]. Although these indicators of developmental milestones are meant to act as resources for parents, the very existence of such guidelines perpetuates normative standards by which parents inevitably compare their infants against a bell curve [13].

Consequently, social media can be seen to extend spaces of comparison among mothers with negative repercussions. For example, research has found that mothers who spent considerable time on social media after giving birth to connect with a broader maternal community and share information about their new infant expressed feelings such as failure, enhanced anxiety, and doubt in relation to their own parenting abilities [13]. Such feelings of insecurity were amplified among mothers exposed to the posts of other mothers, who by comparison appeared to effortlessly return to their prebaby body or better manage their overlapping roles as mothers, partners, and workers [13].

### Objectives

In this paper, we present key findings from a larger qualitative descriptive study [21], where we explored expectant and new parents’ use of digital technologies within the transition to parenting period [1]. This study generated many rich findings and provided novel insights into a relatively understudied area of inquiry as it focused on new parents’ experiences using digital technologies across preconception to postpartum periods to support their early parenting practices [1]. This paper focuses on the findings that identify how new and expectant mothers negotiated tensions of resultant guilt with perceived gains through their use of digital technologies within the transition to parenting period. By using a sociotechnical perspective [22] to interpret the mothers’ experiences, we show how new and expectant mothers’ digital technology use is a complex process that encompasses a wide range of nuances and cannot be simply categorized as entirely “good” or “bad.”

## Methods

### Theoretical Perspective

The current ubiquity of digital technologies makes it difficult to differentiate between one’s internet-based and offline self [15,22] and, by extension, internet-based and offline forms of parenting. Through a sociotechnical lens [22], digital technologies extend and redefine users’ thoughts, emotions, movements, curiosities, interests, and physical bodies into sites of information represented as a digital code. Humans’ navigation of their social world and place within it—their practices of selfhood are then understood as information sites [22].

Although digital technologies have been found to bring moments of relief and companionship to expectant and new mothers [1,4], research that explores the nuances of new and expectant parents’ experiences with guilt alongside perceived gains as they relate to digital technology use throughout the transition to parenting remains scarce. When it comes to understanding mothers’ experiences particularly with digital technologies, scholars note the importance of considering the layered contexts where the domestic and social demands of women’s lives overlap in their roles as mothers, partners, friends, consumers, citizens, and employees [20,23].

### Recruitment and Participants

This study was conducted from 2018 to 2019 in a predominantly urban region of Southwestern Ontario, Canada. Researchers used a purposive sampling strategy along with a snowball sampling technique [24] to recruit adults who had become parents within the past 2 years. Participants were recruited through flyers posted in community spaces with high volumes of new parents, such as day care centers, family health clinics, public health clinics, and children’s play centers. Digital recruitment flyers were also distributed on internet-based buy-and-sell platforms such as Kijiji and social media sites such as Facebook and Twitter. To be eligible to participate in the study, participants had to (1) identify as a new or expectant parent who transitioned to parenting within the last 2 years, (2) identify as aged between 16 and 35 years, and (3) speak fluent English. The age bracket was set to an upper limit of 35 years as the health care needs and risks of women who are of advanced maternal age tend to be different, and they tend to have generationally different levels of education, financial stability, life experience, and emotional maturity. All eligible parents provided written informed consent and were given a US $15 honorarium for their participation before data collection began.

### Ethical Considerations

Ethics approval to conduct this research was granted by the nonmedical research ethics board of Western University (2020-114165-36905). All participants were provided with a letter of information and gave their consent to participate in the focus groups and follow-up interviews. Each participant was assigned a study ID number to protect their anonymity.

### Data Collection and Analysis

Focus groups was chosen as the method of data collection as it honors the coconstruction of knowledge between group members and has been used to engage in meaningful dialogue with new parents [21]. In-person focus groups and follow-up interviews were conducted by members of the interdisciplinary research team. Researchers met participants in pre-agreed locations, including a children’s center, public libraries, and a shelter. Before beginning each focus group, the researchers administered a demographic questionnaire to the parents to capture descriptive characteristics. The main area of inquiry that guided the focus group discussions was parents’ use of and experiences with digital technologies—including pregnancy apps, infant care apps, social media, internet-based support groups, and internet-based health information resources—during their transition to parenting. Participants were asked to reflect on their experiences during the 4 phases of the transition to the parenting period, and probing questions were asked to elicit a deeper discussion of their experiences and interactions with digital technologies. Participants contributed insights based on hearing the responses from others and were prompted by focus group facilitators. All focus groups were audio recorded and transcribed verbatim. Facilitators took field notes throughout the focus groups to capture additional data, specifically nonverbal communication, which could not be documented through digital audio recordings.

Data analysis followed an iterative thematic approach [21,25]. Iterative thematic analysis within the context of qualitative research is a systematic approach to data analysis that is used to identify, analyze, and report patterns or themes within a data set of textual, audio, or visual information [21,25]. This dynamic approach involves multiple rounds of data examination and theme refinement to gain a deeper understanding of the underlying meanings and patterns present within the data set and is particularly useful when examining complex, multifaceted issues [21]. In following this process, each member of the research team individually analyzed the focus group transcripts and field notes to generate an initial coding matrix. Thematic coding was tracked using a tabular matrix with supporting quotes from the transcripts as a semantic guide. Members of the research team iteratively compared their initial codes and emerging insights to coconstruct thematic findings through in-person meetings. Recruitment and data analysis occurred simultaneously and ended once data saturation was achieved when no new themes were generated within focus group discussions or among research team members through iterative analytic discussions [21].

Triangulation and reflexivity were used as mechanisms to ensure rigor and trustworthiness across thematic findings [21,26]. The goal of triangulation within the context of qualitative research is to strengthen the credibility of findings by cross-referencing information from multiple sources or perspectives (ie, researchers) to confirm and strengthen the interpretation of themes or patterns identified in the data. Triangulation is a process that reduces the potential for bias and enhances the reliability of interpretation across multiple data sources [26]. Members of the research team further practiced reflexivity to enhance the rigor and validity of the findings. Reflexivity is a vital component of rigorous qualitative research as it promotes researchers’ self-awareness and active engagement within the research process through acknowledgment and examination of their own beliefs, biases, and assumptions that they bring to their work and how these factors shape their data analysis and interpretative processes. When examining the respective positionalities and lived experience of the researchers within the context of this research, it is important to note that all members of the research team who participated in the data analysis process came from academic backgrounds, including nursing, doula studies, public health, and health professional education, and that most also identified as parents. The research team members’ children ranged in age from 7 to 30 years at the time of data collection. Owing to their diverse experiences within the transition to parenting period at the time of their child or children’s birth, research team members offered different perspectives regarding their personal engagement with digital technologies to support their parenting to bring to the data analysis process. As such, each team member relied on interrelational reflexive practice to guide their dialogues with each other and challenge their own tacit assumptions about using digital technologies within the transition to parenting as codes and eventual themes were identified.

## Results

### Overview

In total, 26 individuals who identified as heterosexual women participated in the study across 10 in-person focus groups (2-4 mothers/focus group). Overall, the age of the participants ranged from 17 to 35 years: 31% (8/26) of the participants were aged ≤20 years, 15% (4/26) of the participants were aged between 21 and 29 years, and 38% (10/26) of the participants were aged between 30 and 35 years. Most participants (18/26, 69%) identified as White, and 12% (3/26) of the participants identified as racialized. In terms of marital status, half of the participants (13/26, 50%) identified as married; 27% (7/26) of the participants identified as single and never married; and 4% (1/26) of the participants identified as separated from their partner. Regarding employment status, one-third of the participants (9/26, 35%) identified as unemployed, 27% (7/26) of the participants identified as a full-time employee, and 12% (3/26) of the participants identified as a part-time employee. Educational background differed among participants at the time the study was conducted; of the 26 participants, 7 (27%) were in the process of completing their secondary school diploma, 1 (4%) held a high-school diploma, 1 (4%) held a community college certificate, 10 (38%) held a university undergraduate degree, and 2 (8%) held a university undergraduate degree as well as a graduate degree. Finally, the socioeconomic status across the participants varied as well; 15% (4/26) of the participants reported a yearly household income of <CAD $20,000 (<US $14,814); 12% (3/26) of the participants reported a yearly household income between CAD $20,000 (US $14,814) and CAD $50,000 (US $37,037); 15% (4/26) of the participants reported a yearly household income between CAD $50,000 (US $37,037) and CAD $99,999 (US $74,073); and 19% (5/26) of the participants reported a yearly household income of >CAD $100,000 (>US $74,074). It is notable that recruitment strategies were inclusive of all genders who become pregnant and a parent; however, all those who expressed interest in the study and enrolled as participants were assigned female at birth.

Overall, participants’ experiences of using digital technologies within the transition to parenting period were analyzed and captured within the overarching theme “balancing the tensions of digital technology use in the transition to parenting” and four subthemes: (1) self-comparison on social media, (2) second guessing parenting practices, (3) communities of support, and (4) trusting intuition over technology.

### Balancing the Tensions of Digital Technology Use in the Transition to Parenting

Participants expressed concerns that their prior digital technology habits were encroaching upon their newfound parenting moments and responsibilities. Navigating smartphone and social media use during infant feeding, sleeping, and bonding time constantly put participants in a conflicted space between feelings of relief and normalcy with respect to their prebaby behaviors and guilt for using it once their baby arrived. For example, they described how they became hyperaware of how frequently they used their smartphone around their new baby:

I’m on it all the time. But not when he—I don’t like to do it when he’s around. So, if he’s busy I’ll do it, but I don’t like to be on it while he’s playing. Like I want to be interactive with him, but I’m still on it as much as I was before.

T1

Participants also recognized how frequently they were on their phone and expressed feelings of guilt, recognizing that not all the time they spent on their device was in service of good motherhood (ie, looking up information or educational sources):

I do feel a little bit, like, I’m on my phone too much, like, I feel like it’s always in my hand or, like, I’m always on it. And, like, not reading, like, you know, it’s not like I’m reading something super informational or educational, right.

T6

There were also times when participants used their smartphone to augment pragmatic and essential parenting responsibilities such as breastfeeding, which caused them to question their technology use habits. The following quote demonstrates how a participant balanced their necessity to stay awake to breastfeed using a smartphone as an aid:

...smartphone scrolling was almost just a way...to stay awake while nursing her.... I sometimes will use it just to like play candy crush or just to kind of stay awake or even if I’m Googling something that she’s doing like it’s probably not the best, but [my smartphone is] always near me.

T7

Participants’ technology use was mirrored back to them by their infants. One participant felt guilty about using her phone as an entertainment source for her toddler after noticing that they picked up on how to use its touchscreen interface and “swipe” notifications out of the way:

If my child is watching a show on my phone and a text message pops there, she’s only 19 months, she can swipe it out of the way. Swipes it out so that it’s off the screen. But I’m like is this my doing that I allow this. And then I feel guilty, but then I’m so exhausted.

T12

These participants undoubtedly questioned the presence of their smartphone and their use of them in front of their infants as they reflected on feelings of guilt and perceived shortcomings in their transition to parenting.

### Self-Comparison on Social Media

Participants described how social media could be both a beneficial tool for social support and a drawback at the same time because of the opportunities it opens for self-comparison:

I think especially in that fragile postpartum period, we’re so vulnerable and we’re so out there, like not feeling good really about where we are.... And, you know, when you feel lonely with your infants, like, I feel like.... You turn...to more social media because it...makes you feel, like, connected in a way but then it makes you more disconnected from where you are...it’s a twisted sort of world.

T12

In terms of self-comparison, the postpartum body was a particular point of comparison for participants, irrespective of their knowledge that social media photos are staged and curated. For example, they described how social media contributed to feelings of body shame:

I think sometimes [social media] is bad for people too like because you are comparing a lot...Like I think it’s good, but it can be bad too because you compare.... I felt like big and ugly...because you’re comparing. You see these like photos, picture perfect pictures that are like set up and the perfect angle and stuff.

T12

Furthermore, participants acknowledged how digital technologies and social media created opportunities to compare themselves to other mothers by reflecting on whether they were meeting cultural expectations of good motherhood:

I have a seven-month-old at home and a three-year old and trying to do all this stuff and just like watching...other people do this and thinking, okay, well maybe I should do that or maybe, you know, I’m not doing enough or I’m not living up to like those expectations. So, I was putting on like unsolicited expectations on myself that weren’t even coming from me, what I wanted and I started kind of like [spiraling].

T12

Participants described balancing the tension between their use of digital technologies as tools to gather parenting information while also recognizing how such technologies can entrap them into making developmental comparison with respect to their infant and other infants:

I feel like I rely on [digital technologies] a lot when it’s like for questions, but I agree with what you [another participant] said about sometimes you’re [likely to] compare to other people if you’re on social media and you see stuff that their kid is doing, maybe not even milestones, but like anything, right. You’re like, oh wow, that’s a– should my kid be doing that or should, I don’t know.

T11

### Second-Guessing Parenting Practices

Although participants remarked how convenient these technologies were in terms of providing them with instantaneous access to health and parenting information, the work of discerning information to inform their parenting practices and infant development was burdensome to participants. Within the transition to parenting period, participants remarked how digital technologies, “definitely guides you...but worries you at the same time and you kind of question everything you’re doing” (T2).

With respect to pregnancy, the participants identified the tension between experiencing relief and self-doubt in their personal application of digital technologies. For instance, a participant used their own home Doppler ultrasound device to listen to their fetus’s heartbeat. Google and YouTube were used as tools to augment the use of the Doppler to search and discern different in utero sounds when they were not sure if what they were hearing was the fetal heartbeat:

...at first, I was like there’s different sounds too right. Like there’s the placenta and then there’s the baby’s heartbeat. So, for me, it was like okay, like YouTubing the sound of like the placenta blood flow versus like a baby’s heartbeat. Like so, I mean I feel like [the doppler] did put me at ease for the most part, maybe when I was having difficulty finding it, then it was like more stressful. And I know like my husband had to like hide it from me from time to time because I was using it too much and then I was like googling like will it like, is it like sound wave safe. Like should I like not be using it. So, I don’t know.

T11

Google was also frequently turned to within the postpartum stage as participants tried to make sense of their babies’ development and determine what was considered normal against the influx of updates and posts of friends’ babies. Participants expressed how digital technologies generated feelings of self-doubt via comparison with others’ posts and their own baby’s developmental progress:

I find it’s hard.... I’m constantly like comparing him to my other friends who have babies around like one of our good friends, [their] baby and him are two days apart and it’s like, yeah, like he’s like really good at holding his head up and but he’s like still kinda wobbling. So, I’m like, is this normal and just googling when do they, when can they do this or when should they be doing this...

T11

### Communities of Support

Participants described how smartphones and social media platforms, such as Facebook, provided crucial opportunities to connect with other mothers to receive support in the form of parenting advice, product information, and tips, and to relieve moments of intense isolation. From Facebook “mommy groups” to texts with friends, informational and social support were notable gains associated with participants’ digital technology use. For example, participants remarked on how comforting it was to “chat” with other parents in Facebook groups and read about their postpartum experiences:

So, there were some [Facebook] groups I joined just to hear other peoples’ stories and things and then others—one I just joined recently and it just talks about anything from like a newborn to toddler.... Just to see other peoples’ experiences and what worked for them, what didn’t work.... And oftentimes, I still kind of make a decision for myself, but it’s nice to know that, okay, someone went through this or someone else had these questions or did this.

T2

Internet-based parenting groups also provided opportunities for the participants to experience social connectedness. For example, participants remarked on how comforting it was to be able to scroll their timelines and stay up to date with others’ lives through their posts even when they were unable to join in such activities. For instance, one participant who was spending most of her time at home and experiencing postpartum depression and feelings of isolation: “looking on Facebook and stuff kinda helped me to just to like, to see what everyone else was doing and it kind of make me feel like I’m still like...connected” (T3). Beyond social media, digital technologies, such as smartphones, also played a crucial role in facilitating a sense of social connectedness and support through SMS text messaging. For example, participants remarked how using their smartphone to connect with others through text made them feel like they were never alone: “it’s so easy to like send a text to someone at three in the morning being like, this is happening or [ask] is this happening to you?” (T11).

### Trusting Intuition Over Technology

Finally, it is important to note that although participants reported the incessant use of digital technologies, they also questioned, challenged, and resisted the pervasive power of digital technologies in the transition to parenting. Participants demonstrated resistance in choosing to trust their own intuition. Rather than using digital technologies that amplified anxieties (instead of soothing them), participants noted specific occasions on which they resisted emerging parental surveillance trends. As one participant noted, they resisted the need to reply on digital technology to alleviate the fears of “unknowing” in parenting:

I’m not gonna put a monitor in there [infant’s room] looking at my kid all the time because I’m just gonna be looking at my kid all the time and freaking out and thinking they have something.... I literally just, I need to trust that they’re okay.

T10

Participants were particularly aware of their capacity to obsess over information (in this case, a real-time video stream of their infant sleeping) and adopted ways they could challenge their own knowledge: “I need to trust that I did a good job. That I tucked them in [that] Some stuff didn’t fly across the room and cover their face” (T10).

## Discussion

### Principal Findings

Participants expressed conflicting feelings toward digital technologies and social media use. On the one hand, they expressed feelings of appreciation and relief, noting these technologies offered “in-the-moment” access to various sources of information and pragmatic support. In line with previous research on pregnancy and digital technology use [2,3,5,27], our findings shed light on the sense of community, timely access to information, reassurance, and validation that digital technologies can provide new parents. However, on the other hand, participants expressed feelings of guilt for using such means as some felt they were not living up to the cultural expectation of good motherhood, which aligns with findings from studies emerging out of psychology [18,19].

The discourse of good motherhood permeated participants’ responses as they described their experiences when encountering “perfect” images of motherhood posted on social media. Although such instances were acknowledged by the participants in this study as performative, the bombardment of these idealized images appeared to impact their self-perception and led to moments of self-critique accompanied by espoused feelings of guilt or anxiety. In addition, participants reported similar experiences of self-critique when consulting commercialized pregnancy and infant care apps as they compared their bodies or infant’s progress against the norms and developmental milestones encoded within a particular app. The implications of these findings such as adverse health outcomes, for example, poor mental health, support recent research that notes the tension between the unrealistic demands of hegemonic motherhood within North America and the inadequate—often harmful—support systems and tools, inclusive of digital technologies such as apps, which actively detract from women’s well-being while strengthening the very patriarchal structures that define these impossible parenting standards in the first place [27,28].

Within the transition to parenting period, expectant and new parents are promised peace of mind, flexibility, efficiency, and connectivity through marketing tactics focused on assurances of a “normal” and “healthy” pregnancy and postpartum experience [5,9]. These strategies are supported by (as they are of benefit to) neoliberal systems where responsibility for health care and specifically maternal and fetal health becomes “self-responsibilitized,” that is, when the responsibility of care falls on the shoulders of individuals [5,9,23]. The pregnant body and the developing fetus become sites of quantifiable information that are entangled and inseparable from the social media platform or app’s information system in which they are being explored and monitored. Under the guise of intimate surveillance, practices of selfhood and care become redefined as practices of information management and data production [9,22].

Although mothers’ use of various social media platforms and apps, such as Facebook, can be seen as a means for them to care for their fetus or new infant by gathering information, advice, and resources, they also perform a form of digital labor by virtue of their user activity across the platform itself [29,30]. In sociotechnical terms [30], the information collected on users’ behaviors, habits, and preferences as they traverse digital spaces—be it social media platforms, apps, or search engines—indirectly produces information capital for app developers and domain hosts to take advantage of, which is why most digital tools and apps are free or offered at a relatively low cost [29]. This unwaged labor mothers are performing across digital technologies, apps, and social media platforms through their day-to-day actions and health work as parents can become exploited by developers for profit [29,31]. For example, mothers’ comments, likes, posts, and shares on Facebook and within the parenting support groups it hosts can be commoditized and sold to third parties for marketing purposes.

Not only are social media platforms and app developers profiting from the sale of users’ (mothers’) information but also the companies that purchase this information may further profit through targeted advertisements and sales of products to the very users (mothers) who accessed such support groups for health information in the first place [29,31]. For health care providers and organizations, this creates opportunities to consider and explore the impact of emerging internet-based health care settings and to question the accountability of predominant stakeholders (health care providers–clinicians, public health organizations, governments, and multinational companies such as Google, Facebook, and Amazon) within the digitally informed health care ecosystem and to question who is driving our health care agenda [29-31].

For mothers in this study, the idealized good mother was illustrated and reinforced through their ubiquitous use of digital technologies to achieve ideal motherhood; yet the promises of good mothering subsumed in the ubiquitous use of digital technologies also contributed to their feelings of guilt and self-doubt, potentially compromising their well-being. Social media platforms and apps can be misleading. Mothers using these digital technologies are at once chasing an unattainable ideal of good motherhood yet perpetuate their use of these apps for surveillance of self and fetus lest they be considered a “bad mother.” From a sociotechnical perspective, we must ask ourselves who benefits from the entanglement of mothers’ and their unborn or newborns’ bodies being digitized into information outside of the transition to parenting period [15,22]. The experiences reported by mothers in this study raise critical questions as to how mothers best use digital technologies to support their transition to parenting in ways that are health enhancing and personally constructive.

### Limitations

Data were collected before the COVID-19 pandemic. As we continue to move beyond the COVID-19 pandemic on a global front, it is important to consider the pandemic’s influence on patterns of digital technology use during pregnancy and parenting given the widespread and necessary reliance on digital technologies to access health information and services. We recognize that our study population was demographically limited in terms of gender identity, racial identity, and age among other equity-deserving groups. All mothers in this study spoke English as their first language and were predominantly White. Future research should seek out the experiences of lesbian, gay, bisexual, transgender, queer, intersex, asexual, allies, and other parents as well as disabled parents within this context as they are underserved and have unacknowledged demographics in many pregnancy-related and parenting-related apps. It would be important to explore whether these parents find community and information in particular places designed with their needs and experiences in mind. We want to acknowledge that parents in different geographic or cultural contexts may experience the importance of the role of these digital technologies in different ways that intersect with the health care and social supports they are receiving or not receiving from other sources dependent on their location.

### Conclusions

Mothers in this study turned to digital technologies for immediate access to health information to support their parenting practices as well as an outlet to connect with other mothers who were in similar stages of the transition to parenting. Performative images of “perfect” motherhood across social media and normative milestones embedded within pregnancy and parenting apps caused the mothers to constantly self-critique. Although digital technologies satisfied these mothers’ informational and social needs, they simultaneously encouraged feelings of guilt and self-doubt that urged them to appropriate whether they were a good mother. By understanding how mothers use technology within the context of pediatric care and the inherent complexity that is bound up within this use, health care providers may work toward providing guidance on reliable health information sources to ensure that parents receive credible information to inform their parenting practices, engaging with internet-based parenting communities to share evidence-based advice and address concerns, and developing mental health and wellness resources that are attuned to the complex nature of technology engagement during this intensive and vulnerable time, all of which can contribute toward improving pediatric outcomes.
